# Evaluation of a Novel eHealth Tool for Pulmonary Rehabilitation in People With Chronic Obstructive Pulmonary Disease: Randomized Controlled Pilot and Feasibility Trial

**DOI:** 10.2196/68195

**Published:** 2025-06-23

**Authors:** Åsa Karlsson, Pernilla Sönnerfors, Sara Lundell, Annika Toots, Karin Wadell

**Affiliations:** 1 Department of Community Medicine and Rehabilitation Umeå University Umeå Sweden; 2 Department of Medicine Solna, Division of Immunology and Respiratory Medicine, Center for Molecular Medicine, Karolinska Institutet Stockholm Sweden; 3 Medical Unit Occupational therapy and Physiotherapy, Womens´s Health and Allied Health Professionals Theme, Karolinska University Hospital Stockholm Sweden

**Keywords:** chronic obstructive pulmonary disease, COPD, telerehabilitation, eHealth, usability testing, user-centered design, pulmonary rehabilitation, eHealth tool, feasibility trial, randomized controlled trial, RCT

## Abstract

**Background:**

There is a growing interest in eHealth solutions to enhance access to and use of pulmonary rehabilitation for people with chronic obstructive pulmonary disease (COPD).

**Objective:**

This study aims to evaluate the feasibility of a novel eHealth tool (Me&COPD) to support pulmonary rehabilitation concerning usability, exercise adherence, intensity, progression, and adverse events. Moreover, this study aims to evaluate clinical outcome measures to prepare for a future larger trial.

**Methods:**

A multicenter, parallel-group randomized controlled pilot and feasibility trial was conducted in 6 primary health care centers. People with mild to severe COPD were recruited by physiotherapists at the included health care centers and randomized either to the intervention group with access to Me&COPD for 3 months or to the control group receiving usual care. The Me&COPD tool comprised audio-visual and written self-management strategies, including an individually tailored home-based exercise program and interaction with a physiotherapist. The exercise program was prescribed in a face-to-face meeting with a physiotherapist, and thereafter it was regularly reviewed and adjusted through the eHealth tool. The primary outcome, usability, was self-assessed at intervention completion in the intervention group and among participating physiotherapists (n=7) using the Swedish version of the Mobile Health App Usability Questionnaire (S-MAUQ). In addition, use data on exercise adherence, intensity, and progression and adverse events were exported from the eHealth tool. Clinical outcomes, assessed by blinded assessors at baseline and 3 months in the intervention and control groups, included exercise capacity, balance, physical activity level, COPD-related symptoms, and health-related quality of life. Descriptive statistics were used for analysis.

**Results:**

In total, 22 participants (women: n=12, 55%), aged 72.3 (SD 8.4) years on average, were included in the intervention (n=15) and control (n=7) groups. The mean overall S-MAUQ scores out of 7 (highest possible usability) were 4.4 (SD 1.5) for participants and 4.5 (SD 1.2) for physiotherapists. Among the subscales, the highest score was assigned to *usefulness* among participants (S-MAUQ: mean 4.9, SD 1.3) and physiotherapists (S-MAUQ: mean 5.1, SD 1.7). No severe adverse events were registered, although exercise adherence, intensity, and progression evaluation were limited by incomplete exercise session registration. The test procedures and the clinical outcome measures used were found to be feasible for the participants and the assessors.

**Conclusions:**

The novel eHealth tool, Me&COPD, seemed feasible in terms of safety and had acceptable usability among people with COPD and participating physiotherapists. Usability may be improved by better organization of the information and simplification of the exercise diary to enable collection of data on exercise adherence, intensity, and progression through the eHealth tool. The test procedures seemed feasible, although the recruitment process needs further consideration. The effectiveness of the intervention remains to be evaluated in a future larger trial.

**Trial Registration:**

ClinicalTrials.gov NCT05086341; https://clinicaltrials.gov/study/NCT05086341

## Introduction

### Background

Chronic obstructive pulmonary disease (COPD) is a complex and heterogeneous disease that not only involves airflow limitation but also multiple extrapulmonary manifestations, such as muscle dysfunction, decreased exercise capacity and balance, cardiovascular disease, fatigue, and depression [1]. Fortunately, there is an established nonpharmacological intervention, pulmonary rehabilitation (PR), that can address these systemic consequences and improve the health of people with COPD [2]. PR is defined as a comprehensive, individualized intervention including but not limited to physical exercise, education, and behavioral change that aims to improve the individual’s physical and psychological condition and promote long-term adherence to health-enhancing behaviors [3]. There is conclusive evidence of the positive effects of PR on exercise capacity, symptoms, health-related quality of life, and reduced health care use [2-5]. The intervention has proven to be cost-effective in different settings [6]. Despite strong recommendations in national as well as international guidelines [3,4,7-9], the accessibility of PR for people with COPD is limited [10]. According to Desveaux et al [10], the available PR programs in 7 different countries could only serve less than 1.2% of the population with COPD in these countries, with Sweden having the lowest annual capacity of 0.2%. In addition, central barriers to participation in PR are distance to PR centers, limited knowledge of benefits, and fluctuating health status [11,12], emphasizing a need for novel strategies to provide person-centered care and improve access to PR among people with COPD [13].

eHealth solutions, that is, the use of information and communication technologies in health services [14], such as telerehabilitation, represent a promising alternative strategy to improve access to and uptake of PR [15,16]. The COVID-19 pandemic has further highlighted the need for rehabilitation services provided at a distance and accelerated their development. Telerehabilitation for people with COPD, provided at home or at an outpatient health care facility, has been shown to be safe with comparable effects on exercise capacity, quality of life, and symptoms as in-person, center-based PR [15,17,18]. Furthermore, home-based telerehabilitation interventions have been reported to increase the likelihood of program completion [15] and be more effective than usual care in terms of improvements in dyspnea [18] and health status [18,19]. However, to be able to implement an eHealth solution in clinical practice, it is crucial that the intervention is accepted by those who are going to use it and the intervention fits in the context where it will be implemented [20]. A previous study reported that the users, that is, people with COPD, relatives, health care providers (HCPs), and a patient organization representative, had a positive attitude toward using an eHealth tool to support self-management of physical activity and exercise training [21]. However, they expressed that eHealth solutions should be seen as a complement to already existing rehabilitation options. Furthermore, Tsai et al [22] reported that a home-based exercise program using real-time videoconferencing technology was well accepted by the study participants, and the informants taking part in a supervised telerehabilitation program expressed that the intervention provided autonomy support [23]. Moreover, eHealth tools have been voiced to support and enhance self-management of the disease [24,25]. However, it has also been found that using digital technology is not suitable for everyone, such as for individuals who lack motivation, are fearful of new information, and are not comfortable with IT [26]. This was emphasized in the study by Cox and Holland [27] who concluded that one size does not fit all, and more research is needed on different models of telerehabilitation in people with COPD to reach evidence-based consensus and increase possibilities for individualization [16,28].

To promote health, provide individualized treatment, and increase access to evidence-based treatment for people with COPD in Sweden, we developed an eHealth tool for PR, Me&COPD (Swedish: Min KOL). The tool was developed in a structured cocreation process with intended end users [29], which was not common in the development of previous eHealth tools [28]. The use of the cocreation process may be beneficial for the implementation of the intervention [30] and result in more problem-based research, increased contextual relevance, and greater impact [31]. The content of Me&COPD aligns with the international guidelines for PR in COPD [32], the national guidelines for COPD care in Sweden [4], and with reported requirements and needs of people with COPD and HCPs [21,33,34]. The Me&COPD tool was built in collaboration with health care region Västerbotten on 1177 Care guide—Support and Treatment (Swedish: 1177 Vårdguiden—Stöd och Behandling) platform, an established, national medical information system that provides a platform for various internet-based support and treatment programs. However, none of the platform’s existing programs have targeted PR in people with COPD.

### Aims

The aim of this trial was to evaluate the feasibility of Me&COPD in terms of usability, exercise adherence, intensity, progression, and adverse events. An additional aim was to evaluate clinical outcome measures to prepare for a future larger trial.

## Methods

### Trial Design

We conducted a multicenter, parallel-group randomized controlled pilot and feasibility trial, which was reported according to the CONSORT (Consolidated Standards of Reporting Trials) statement for pilot- and feasibility trials [35], the eHealth checklist [36], and the TIDieR (Template for Intervention Description and Replication) checklist [37]. The trial was prospectively registered at ClinicalTrials.gov (ID NCT05086341).

### Ethical Considerations

The trial received ethics approval from the Swedish Ethical Review Authority (Dnr: 2020-01693 and 2021-03538) and was conducted according to the Declaration of Helsinki. Participants were informed about the trial by telephone. If oral consent was obtained, a package encompassing the details of the trial procedures, their right to withdraw from the study without prejudice, a consent form, and baseline questionnaires was posted to the participants and collected at the baseline assessment. All participants provided written informed consent to participate in this trial. To ensure confidentiality of the participants, data were stored encrypted at a secure server at the Umeå University with access restricted to the research team. The security solution, Cryptshare (Pointsharp Secure Information Exchange solution), was used for secure data transfer between the participating health care regions and the researchers at the Umeå University. Participants were offered reimbursement for their travel expenses, but no other monetary compensation was offered.

### Settings, Recruitment, and Participants

The trial was conducted between November 2021 and June 2023 at 6 primary health care centers (PHCs). PHCs are the first point of access to health care for many, especially those with chronic diseases such as COPD. PHCs offer services, including assessment, treatment, prevention, and rehabilitation without the need for a referral. Most PHCs in Sweden are multiprofessional, including experts such as general practitioners, nurses, occupational therapists, and physiotherapists. The PHCs in this trial were situated in big and medium-sized cities in central and northern parts of Sweden. Publicly funded as well as private alternatives were included. PHCs in 2 health care regions were included at trial commencement. Due to the low recruitment rate, a third health care region was included, and the trial period was extended by 6 months. To facilitate recruitment of PHCs into the trial, 1 member of the research group worked exclusively with approaching staff at eligible PHCs, that is, physiotherapists experienced in COPD, their managers, and COPD nurses using personal letters, emails, and telephone. Furthermore, the research group spread information about the trial by participating in digital meetings with the regional administrations and COPD-related education events for HCPs. Potential participants, that is, adults with COPD who were in a stable condition [32], were identified by physiotherapists at the participating PHCs using their patient lists. The physiotherapists asked for their approval to let a researcher contact them for trial information and delivered the intervention for those who were randomized to the intervention group.

Inclusion criteria were (1) a confirmed diagnosis of COPD, (2) the ability to read and understand Swedish, and (3) the absence of severe comorbidity that could be considered as the main contributing factor for limitation in physical activity. The trial had no definite inclusion criteria for computer literacy, and if a participant did not have access to a computer, tablet, or smartphone, there were tablets available to borrow from the research group. In case of a COPD exacerbation, the participant had to wait 6 weeks from the start of pharmacological treatment before being eligible for the trial.

### Randomization

Following baseline assessment, participants were randomized either to the intervention (Me&COPD) or the control group (usual care) in a 2:1 allocation ratio with stratification for sex. A computer-generated randomization list was used, and the results were stored in sealed envelopes. A researcher, not involved in data collection, administered the randomization and assigned participants to interventions.

### Intervention

**Figure 1:**
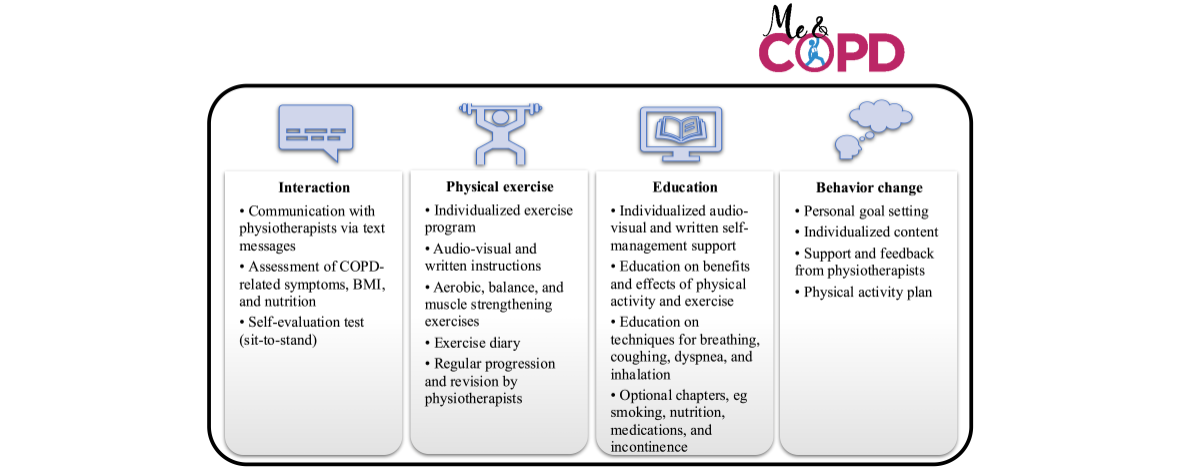
An overview of the content and features of the eHealth tool Me&COPD. COPD: chronic obstructive pulmonary disease.

In addition to usual care, participants in the intervention group had access to the Me&COPD tool during the 3-month intervention period. The intervention was provided on the 1177 Support and Treatment platform, which was accessed by the participants through a secure log-in using a national electronic identification system. The intervention included components such as a physical activity plan, an individualized exercise program, and educational texts and films to support self-management strategies. Components important for behavioral change, such as personal goal setting and feedback, were also included [38]. The exercise program comprised warm-up exercises and 5 muscle strength or endurance exercises, targeting the shoulders, thighs, and calves, as well as balance and aerobic exercises, all with an individually adapted degree of difficulty and intensity and emphasis on safe performance. The exercise program was to be performed at least two times per week across the intervention period, that is, a minimum of 24 sessions. To monitor exercise intensity, the participants were instructed to report their exercise experience and symptoms (dyspnea and muscle fatigue ratings) for each exercise on the Borg CR10 scale [39] in an exercise diary after each session. If the participants had not accessed Me&COPD in the last 7 days, they received a reminder (email or SMS text message) to log in to the eHealth tool. An overview of the content and features of the intervention is presented in Figure 1.

During an initial in-person meeting at the PHC with their physiotherapist, participants received detailed information on how to use Me&COPD as well as a booklet about the log-in procedure of the 1177 Support and Treatment platform. In addition, the individualized exercise program and physical activity plan were set up. The remaining part of the intervention was performed in the participants’ homes with the possibility to communicate with the physiotherapists through the eHealth tool. During the intervention period, the physiotherapists were advised to log in to Me&COPD regularly, that is, at least once a week to answer any messages from the participants and every other week to review and adjust or further develop the exercise program when needed, based on the information provided from participants’ symptom assessments in the exercise diary. The physiotherapists were recommended to shift from continuous- to interval-based aerobic training after 4 weeks and prescribe muscle strength exercises no longer than 4 weeks before shifting to muscle endurance exercises for the remainder of the intervention, if possible [40,41]. The participants were instructed to use ordinary contact paths for health care in case of acute illness. If a COPD exacerbation occurred during the intervention, access to Me&COPD could be extended by up to 2 weeks. The participants were allowed to take part in other physical activities or interventions involving physical activity during the trial period.

Technical support was available throughout the intervention period for both participants and physiotherapists via the 1177 Support and Treatment platform and the study coordinator. Before study commencement, the physiotherapists also received a digital training session with the study coordinator and a representative from the 1177 Support and Treatment platform as well as a detailed, written user manual and practical exercises to learn how to use Me&COPD. The content of the intervention was not changed during this trial.

### Control

All participants in the control group received the usual care alone. Usual care is recommended to include (but is not restricted to) the use of long-acting anticholinergics and long-acting β2-agonists with 24-hour duration and support for smoking cessation, physical activity and exercise, self-management, and nutrition [4,7]. There were no restrictions on those allocated to the control group to participate in other physical activities or physical activity interventions during the trial period.

### Outcomes

All participants were invited to in-person test visits at baseline and at the end of the intervention, that is, at 3 months, at a university or a health care facility. Two researchers (physiotherapists) blinded to group allocation performed the assessments. Usability was the primary outcome of the trial, measured at intervention completion in the intervention group and in participating physiotherapists at the included PHCs, using the Mobile Health App Usability Questionnaire (MAUQ; the interactive version for patients and HCPs) [42]. The patient versions of the questionnaire have been found to have strong validity and reliability [42]. The questionnaire comprises 21 statements on a 7-point Likert scale ranging from 1 (strongly disagree) to 7 (strongly agree), with an additional “not applicable” option available for all items (maximum 147 points). The scale can be divided into 3 subscales: *ease of use and satisfaction* (items 1-8), *system information arrangement* (items 9-14), and *usefulness* (items 15-21). The average of the responses to all statements is calculated for the entire scale as well as for each subscale where a mean score of 7 indicates the best possible usability. In this trial, we used a Swedish version of the MAUQ (S-MAUQ) where the patient version had space in the end for comments. The questionnaire was translated into Swedish using a forward-backward translation procedure by native speakers in the respective language [43]. Two of the researchers translated the English version to Swedish, and 1 independent person without a medical background performed a back translation to English. The research group reached consensus on the final version after discussions and review by a representative at the unit for IT support and system development at the Umeå University. To evaluate exercise adherence, intensity, progression, and adverse events, use data were exported from the eHealth tool.

Clinical outcomes, assessed using valid and reliable tests and questionnaires for people with COPD, were collected to prepare for a future larger trial. Exercise capacity was measured by the 6-minute walk test [44], the 1-minute sit-to-stand test [45], and the unsupported upper limb exercise test [46]. Balance was measured by the timed up and go test [47]. Physical activity level was self-reported using a questionnaire from the Swedish National Board of Health and Welfare [48]. The mean number of steps per day was objectively measured over 7 consecutive days using accelerometers validated in people with COPD (DynaPort; McRoberts BV). The assessors attached the accelerometer at the end of the in-person test visit at baseline and at the 3-month follow-up, and the measurement started later the same day. Measurements with ≥4 valid weekdays were included, that is, weekends and weekdays with <8 hours of daytime wear time of the accelerometer were excluded [49]. In addition, COPD-related symptoms were measured using the Modified Medical Research Council dyspnea scale [50] and the COPD Assessment Test [51]. Health-related quality of life was measured by the St George’s Respiratory Questionnaire [52] and health status by EQ-5D [53]. The frequency of COPD-related health care contacts during the trial was reported by the participants.

Baseline descriptive assessments included a lung function test (spirometry) according to guidelines [54]. Demographic and anthropometric measurements included age, sex, height, weight, and BMI. All participants were asked to report on living conditions, educational level, employment, smoking status, medications, comorbidities, and COPD-related health care contacts in the past year. Descriptive characteristics of the participating physiotherapists included age, number of years in the profession, and educational level.

### Sample Size and Data Analysis

Because this was a pilot and feasibility trial, no formal sample size calculation was performed. The sample size estimation was based on an expected medium standardized mean difference (medium target effect size of 0.3≤d<0.7) between the intervention and control groups on the 6-minute walk test in an 80% powered future definitive randomized controlled trial, suggesting that about 10 participants per group were needed [55,56]. However, to gain more knowledge about the feasibility of the intervention, we aimed to allocate 20 participants to the intervention group and 10 to the control group (30 participants in total). Data were analyzed according to the intention-to-treat principle using available data from all participants, according to their original allocation and regardless of level of attendance. Descriptive statistics with estimates were used for analysis [35]. Data are reported as means, SD, or frequencies (percentages) in this paper. For clinical outcomes with continuous data, a paired 2-tailed *t* test was used to describe within-group changes (mean and SD) between baseline and 3 months. An independent sample *t* test was used to determine if the mean changes were different between the groups (mean and 95% CI). Statistical analyses were conducted using SPSS Statistics (version 29.0.1.0; IBM Corp). The outcome usability was also reported narratively based on written comments from the participants.

## Results

### Participant Characteristics

**Figure 2:**
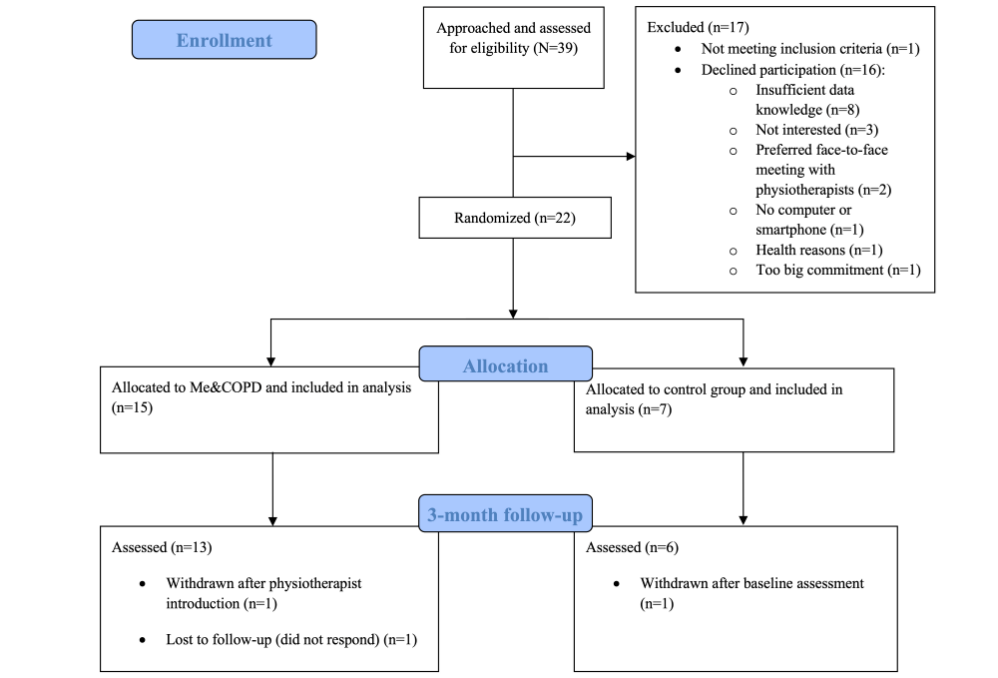
CONSORT (Consolidated Standards of Reporting Trials) participant flow diagram.

The recruitment rate was slow, and despite a 6-month trial extension and inclusion of an additional health care region, the intended sample size was not reached. Of the 39 approached eligible participants, 22 (56%) were included and randomized to either the intervention (n=15, 68%) or control (n=7, 32%) group (Figure 2).

The participants lived in central (16/22, 73%) and northern (6/22, 27%) parts of Sweden. One participant in the intervention group was introduced to Me&COPD by the physiotherapist but did not use the tool during the intervention period, although the participant was included in the analysis and assessed at the 3-month follow-up in accordance with the intention-to-treat approach. Participants’ characteristics are shown in Table 1. On average, they were aged 72.3 (SD 8.4) years; 55% (12/22) were women. The disease severity ranged from mild to severe according to the Global Initiative for Chronic Obstructive Lung Disease [32].

Over 70 PHC managers in the included health care regions were personally contacted, of whom 6 agreed to participate in the trial. Reasons for refusal included an insufficient number of patients with COPD, high workload, or not being connected to the 1177 Support and Treatment platform. In total, 7 physiotherapists, 5 (71%) women and 2 (29%) men, working at the participating PHCs, were included in the trial and answered the S-MAUQ questionnaire for HCPs. On average, they were aged 47.7 (SD 17.6) years and had worked for 17.6 (SD 10) years as a physiotherapist.

**Table 1:** Baseline characteristics of the included adults with chronic obstructive pulmonary disease in the randomized controlled pilot and feasibility trial (N=22).

Characteristic			Total		Intervention (n=15)		Control (n=7)
Age (y), mean (SD)			72.3 (8.4)		74.5 (7.4)		67.6 (8.9)
**Sex, n (%)**							
	Female	12 (55)		9 (60)		3 (43)	
	Male	10 (45)		6 (40)		4 (57)	
Living alone, n (%)			10 (45)		8 (53)		2 (29)
**Employment status, n (%)**							
	Employed	4 (18)		2 (13)		2 (29)	
	Unemployed	1 (5)		0 (0)		1 (14)	
	Retired	17 (77)		13 (87)		4 (57)	
BMI (kg/m^2^), mean (SD)			25.6 (4.6)		25.7 (5)		25.2 (4)
FEV_1_^a^ (L), mean (SD)			1.7 (0.6)		1.7 (0.5)		1.8 (0.8)
FEV_1_, % predicted, mean (SD)			65.7 (20.9)		66.8 (20.4)		63.3 (23.4)
FEV_1_/FVC^b^, mean (SD)			0.6 (0.1)		0.6 (0.1)		0.6 (0.2)
**GOLD^c^ grades, n (%)**							
	Grade 1	6 (27)		4 (27)		2 (29)	
	Grade 2	10 (45)		7 (47)		3 (43)	
	Grade 3	6 (27)		4 (27)		2 (29)	
	Grade 4	0 (0)		0 (0)		0 (0)	
**GOLD^d^ groups, n (%)**							
	Grade A	5 (23)		5 (33)		0 (0)	
	Grade B	11 (50)		5 (33)		6 (86)	
	Grade E	6 (27)		5 (33)		1 (14)	
**Exacerbations in the previous year, n (%)**							
	1	3 (14)		3 (20)		0 (0)	
	2-3	4 (18)		3 (20)		1 (14)	
	≥4	1 (5)		1 (7)		0 (0)	
**Smoking status, n (%)**							
	Former	21 (95)		15 (100)		6 (86)	
	Current	2 (9)		0 (0)		2 (29)	
Pack year^e^, mean (SD)			20.7 (10.4)		23.3 (9.3)		14.2 (10.7)
**Diagnoses (current or former), n (%)**							
	Diabetes	1 (5)		1 (7)		0 (0)	
	Hypertension	10 (45)		8 (53)		2 (29)	
	Stroke	2 (9)		1 (7)		1 (14)	
	Heart disease^f^	5 (23)		5 (33)		0 (0)	
	Osteoporosis	4 (18)		2 (13)		2 (29)	
	Depression and anxiety	2 (9)		1 (7)		1 (14)	
	Sleep apnea	6 (27)		4 (27)		2 (29)	
	Cancer	7 (32)		5 (33)		2 (29)	
Daily steps^g^ (n=21), mean (SD)			6704 (2512)		6525 (2130)		7151 (3494)
Daily steps, all valid days^h^ (n=21), mean (SD)			6645 (2830)		6513 (2544)		6975 (3707)

^a^FEV_1_: predicted forced expiratory volume in 1 second.

^b^FEV_1_/FVC: ratio between FEV_1_ and forced vital capacity.

^c^GOLD: Global Initiative for Chronic Obstructive Lung Disease.

^d^Values are calculated according to the Chronic Obstructive Pulmonary Disease Assessment test score.

^e^Pack year is the equivalent of smoking 1 pack of cigarettes per day for 1 year.

^f^Diseases include myocardial infarction, angina pectoris, or heart failure.

^g^Measurements of ≥4 valid weekdays with at least 8 hours of daytime wearing time.

^h^For comparison between studies according to the international task force on physical activity [49].

### Usability

**Figure 3:**
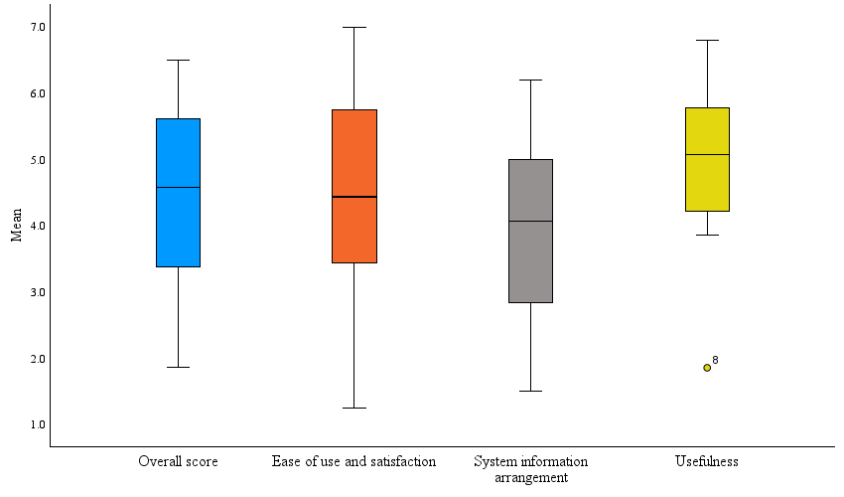
Boxplots showing the intervention groups’ overall score on the Mobile Health App Usability Questionnaire and scores of the subscales ease of use and satisfaction, system information arrangement, and usefulness at the end of the trial. The boxplot whiskers indicate the highest and lowest values, respectively.

A total of 80% (12/15) of the participants in the intervention group answered the S-MAUQ questionnaire at the 3-month follow-up. The mean overall score was 4.4 (SD 1.5) out of 7; that is, responses were overall neutral, with scores ranging between 1.9 and 6.5. The average overall score and the scores for the subscales are illustrated in Figure 3.

Among the subscales, the highest score was assigned to *usefulness* (Table 2). The statements that received the highest scores were that the app had been useful for participants’ health and well-being and improved interaction with the physiotherapist and that participants felt confident to communicate via the eHealth tool. The mean score for each statement is presented in Table 2.

Some (7/12, 58%) participants made comments on the S-MAUQ questionnaire regarding their experience of using the Me&COPD eHealth tool. The positive aspects were related to the subscale *usefulness* where participants appreciated having a flexible exercise schedule and felt confident about safety. The negative aspects were related to the subscales *ease of use and satisfaction* and *system information arrangement* and were concerned with how information was organized and also that some functionalities of the eHealth tool were unsatisfactory. A summary of the participants’ comments is shown in Textbox 1.

**Figure 4:**
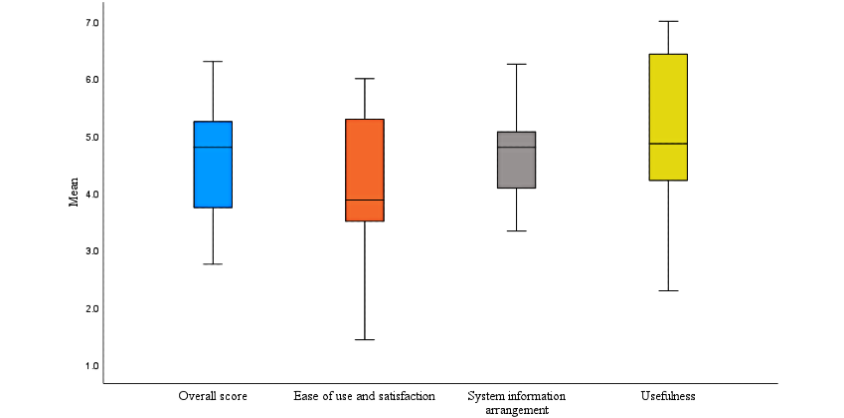
Boxplots showing the physiotherapists’ overall score on the Mobile Health App Usability Questionnaire and scores of the subscales ease of use and satisfaction, system information arrangement, and usefulness at the end of the trial. The boxplot whiskers indicate the highest and lowest values, respectively.

Among physiotherapists, the mean overall score on the S-MAUQ was 4.5 (SD 1.2) out of 7; that is, responses were overall neutral, with scores ranging between 2.8 and 6.3. The average overall score and the scores for the subscales are illustrated in Figure 4.

**Table 2:** The intervention groups’ scores on the Swedish version of the Mobile Health App Usability Questionnaire (S-MAUQ) for the subscales and individual statements at the end of the trial (n=12).

S-MAUQ statements		Scores, mean (SD)
**Ease of use and satisfaction**		4.4 (1.7)
	The app was easy to use.	4.1 (2.2)
	It was easy for me to learn to use the app.	4.3 (2.1)
	I like the interface of the app.	4.2 (2.1)
	The information in the app was well organized so I could easily find the information I needed.	4.2 (1.6)
	I feel comfortable using this app in social settings (n=10).	4.1 (2.6)
	The amount of time involved in using this app has been fitting for me.	4.8 (2)
	I would use this app again.	4.8 (2.3)
	Overall, I am satisfied with this app.	4.6 (1.8)
**System information arrangement**		3.9 (1.5)
	Whenever I made a mistake using the app, I could recover easily and quickly (n=9).	2.6 (1.7)
	This mHealth app provides an acceptable way to receive health care services (n=11).	4.4 (1.8)
	The app adequately acknowledged and provided information to let me know the progress of my action.	3.5 (2.2)
	The navigation was consistent when moving between screens.	3.6 (1.9)
	The interface of the app allowed me to use all the functions (such as entering information, responding to reminders, viewing information) offered by the app.	4.4 (1.4)
	This app has all the functions and capabilities I expected it to have.	4.4 (1.9)
**Usefulness**		4.9 (1.3)
	The app would be useful for my health and well-being.	5.2 (1.4)
	The app improved my access to health care services (n=10).	4.2 (1.8)
	The app helped me manage my health effectively (n=10).	4.3 (1.6)
	The app made it convenient for me to communicate with my healthcare provider. (n=11).	5.2 (1.9)
	Using the app, I had many more opportunities to interact with my healthcare provider (n=11).	5.2 (1.5)
	I felt confident that any information I sent to my provider using the app would be received (n=11).	5.4 (1.5)
	I felt comfortable communicating with my healthcare provider using the app (n=11).	4.9 (1.9)

**A summary of user experiences in the intervention group at the end of the trial.**

- An advantage to have the opportunity to choose when and where the exercise program should be performed (n=1)
- Felt safe to use (n=1)
- Required a certain degree of computer skills (n=2)
- Unintuitive interface, making it difficult to find the desired content (n=5)
- The exercise diary was not satisfactory, not clear enough, and lacked the possibility to retrospectively register exercises (n=3)
- Unhandy to have to log in to the 1177 platform to access the tool (n=2)
- The in-person meeting with the physiotherapist, where the eHealth tool was introduced, was not properly structured (n=2)

Similar to the participants, the physiotherapists assigned the highest score to the *usefulness* subscale (Table 3). Statements that received the highest scores were regarding more opportunities to interact with the patients, improved access to and delivery of health care services, and the app being useful for their health care practice (Table 3). Some statements had a low response rate, particularly statement 5, because 86% (6/7) of the physiotherapists had chosen the “not applicable” option. In sensitivity analyses where the questions with the lowest response rate (questions 5 and 9) were removed, the results remained essentially the same.

**Table 3:** The physiotherapists’ scores on the Swedish version of the Mobile Health App Usability Questionnaire (S-MAUQ) for the subscales, and individual statements at the end of the trial (n=7).

S-MAUQ statements			Scores, mean (SD)
**Ease of use and satisfaction**			4.1 (1.5)
	The app was easy to use.	4.1 (1.7)	
	It was easy for me to learn to use the app.	4.6 (1)	
	I like the interface of the app (n=6).	4 (2.3)	
	The information in the app was well organized so I could easily find the information I needed.	4.3 (1.8)	
	I felt comfortable using this app in social settings (n=1).	3^a^	
	The amount of time involved in using this app has been fitting for me (n=6).	3.8 (1.7)	
	I would use this app again.	4.4 (2.6)	
	Overall, I am satisfied with this app.	3.9 (2)	
**System information arrangement**			4.7 (1)
	Whenever I made a mistake using the app, I could recover easily and quickly (n=4).	4 (0.8)	
	This mHealth app provides an acceptable way to deliver health care services (n=6).	5.2 (2.2)	
	The app adequately acknowledged and provided information to let me know the progress of my action (n=6).	4.8 (1.3)	
	The navigation was consistent when moving between screens.	4 (1.4)	
	The interface of the app allowed me to use all the functions (such as entering information, responding to reminders, viewing information) offered by the app (n=5).	3.8 (0.8)	
	This app has all the functions and capabilities I expected it to have.	5 (1.3)	
**Usefulness**			5.1 (1.7)
	The app would be useful for my health care practice.	5 (2.8)	
	The app improved my access to delivering health care services.	5.4 (2.2)	
	The app helped me manage my patients’ health effectively.	4 (2.4)	
	The app made it convenient for me to communicate with my patients (n=6).	4.8 (1.2)	
	Using the app, I had many more opportunities to interact with my patients. (n=6)	5.8 (1.2)	
	I felt confident that any information I sent to my patients using the app would be received (n=6).	4.5 (1.6)	
	I felt comfortable communicating with my patients using the app (n=6).	4.7 (1.9)	

^a^No SD is reported as only 1 physiotherapist responded to this item.

### Exercise Adherence, Intensity, Progression, and Adverse Events

To evaluate exercise adherence, intensity, progression, and adverse events, data were exported from the eHealth tool. In total, data were exported for 12 participants from 4 of the 6 PHCs. Data showed that the participants had a mean of 11.2 (SD 10.3) registered exercise sessions of the intended 24 sessions, with a range between 1 and 28 registered sessions. During the trial, the physiotherapists reported difficulties faced by the participants in registering their exercise sessions in the exercise diary. The limited number of registered exercise sessions meant that evaluation of exercise intensity and progression was not adequate to perform. No falls or other severe adverse events were reported in relation to the exercise program. A total of 5 (42%) participants described single, minor, or transient adverse events, such as joint-related pain (hip, knee, ankle, and wrist), cough, pain from the torso, and breathlessness more than usual in connection with exercise. In addition, 2 (17%) participants commented on previous shoulder and hand impairments being an obstacle to the upper limb exercises. During the baseline assessment, 1 (8%) participant with a previous hip injury experienced increased hip pain. The pain negatively impacted the participant’s daily life, decreased the walking distance, and limited leg and cardio training during the trial period.

### Clinical Outcome Measures

The test procedures and the clinical outcome measures used were found to be feasible for the participants as well as the assessors. The clinical outcomes for the groups are presented in Table 4. In total, 3 (16%) of 19 participants, 2 (33%) in the control and 1 (8%) in the intervention group, had a COPD exacerbation between baseline and the 3-month follow-up, although hospitalization was not required neither was the intervention extended for the participant in the intervention group. The participants reported few additional COPD-related health care contacts during the trial. In total, 23% (3/13) of the participants in the intervention group received a follow-up from a COPD team, a physician, or a nurse specialized in COPD. Moreover, 17% (1/6) of the participants in the control group participated in structured COPD-related training sessions.

**Table 4:** Clinical outcomes at baseline and at the 3-month follow-up for the intervention and control groups.

Clinical outcomes			Me&COPD, mean (SD)						Control, mean (SD)							Between-group difference, mean (95% CI)	
			Baseline (n=15)		3-month follow-up (n=13)		Within-group difference (n=13)		Baseline (n=7)		3-month follow-up (n=6)		Within-group difference (n=6)				
**Tests, mean (SD)**																	
	6MWT^a^ (m)	450.3 (82.7)		465.3 (88)^b^		23.2 (62.9)^b^		502.9 (107.6)		491.2 (125.5)		–10.2 (23.2)		–33.4 (–90.4 to 23.5)^b^			
	60-second STS^c^	19.3 (7.1)		21 (6.8)^b^		2.1 (4.6)^b^		20.1 (2.4)		20.5 (3.1)		0.0 (2.8)		–2.1 (–6.4 to 2.3)^b^			
	TUG^d^, (s)	10.1 (1.8)		9.8 (2.5)^b^		–0.1 (1.5)^b^		10.4 (2.8)		8.5 (2.9)		–2.2 (1)		–2.1 (–3.5 to –0.6)^b^			
	UULEX^e^ (s)	501.7 (153.6)		453.3 (197)^b^		–43.8 (231.6)^b^		487.4 (122.4)		518.8 (162.4)		25.5 (88.9)		69.3 (–140.9 to 279.5)^b^			
	Daily steps^f^	6525.1 (2129.8)		5497.3 (2507.6)		–874.1 (2488.8)		7150.7 (3493.6)^b^		7226.4 (2215.5)		75.8 (3080.9)^b^		949.9 (–1837.2 to 3737.1)			
**Questionnaires**																	
	PA^g^ level, mean (SD)	10.7 (3.6)		11 (4.3)		0.1 (4.3)		9.3 (5.2)		12 (4)		2.5 (6.7)		2.4 (–2.9 to 7.8)			
	≥150 minutes of PA per week, n (%)	6 (40)		6 (46)		—^h^		2 (29)		3 (50)		—		—			
	CAT^i^, mean (SD)	12.6 (5.3)		13.2 (4.7)		0.0 (4.5)		17 (4.5)		19.5 (8.5)		2.3 (6.8)		2.3 (–3.2 to 7.8)			
	CAT ≥10, n (%)	9 (60)		10 (77)		—		7 (100)		6 (100)		—		—			
	mMRC^j^, mean (SD)	1.4 (0.6)		1.5 (0.8)		0.0 (0.6)		1.29 (0.8)		1.83 (0.8)		0.7 (0.8)		0.7 (–0.0 to 1.4)			
	mMRC ≥2, n (%)	5 (33.3)		4 (30.8)		—		3 (42.9)		4 (66.7)		—		—			
	SGRQ^k^, mean (SD)	25.3 (10.4)		26.6 (11.8)		0.6 (4.5)		32.8 (13.8)		37.3 (10)		5.5 (10.6)		4.9 (–6.3 to 16)			
	EQ-5D, health status, mean (SD)	61.7 (17)		68.8 (13)^b^		10 (15.4)^b^		63.3 (12.6)		68.7 (13.4)		6.5 (8.7)		–3.5 (–18 to 11)^b^			

^a^6MWT: 6-minute walk test.

^b^One participant was missing.

^c^STS: sit-to-stand test.

^d^TUG: timed up and go test (higher values indicate worse balance).

^e^UULEX: unsupported upper limb exercise test (higher values indicate better upper limb function).

^f^Measurements with ≥4 valid weekdays and with at least 8 hours of daytime wearing time were included.

^g^PA: physical activity (physical activity level according to the Swedish National Board of Health and Welfare indicator questions; values of ≥11 correspond to ≥150 minutes of at least moderate physical activity per week.

^h^Not applicable.

^i^CAT: Chronic Obstructive Pulmonary Disease Assessment Test (higher values indicate greater impact of chronic obstructive pulmonary disease).

^j^mMRC: Modified Medical Research Council dyspnea scale (higher values indicate more dyspnea).

^k^SGRQ: St George’s Respiratory Questionnaire (higher values indicate a worse state of health).

## Discussion

### Principal Findings

This trial is the first to evaluate the feasibility of a novel eHealth tool for home-based PR called Me&COPD that has been developed in a structured cocreation process with end users [29]. The primary finding concerned the usability of the tool, where people with COPD and physiotherapists in PHCs rated overall usability as acceptable. The subscale *usefulness* was assigned the highest scores by both people with COPD and physiotherapists, supporting the notion that Me&COPD may be a suitable alternative strategy to provide PR in people with COPD. However, the trial results did indicate that the exercise diary was underused and needs further development in order to collect data on exercise adherence, intensity, progression, and adverse events. The clinical outcome measures used were found to be feasible.

### Interpretation of the Findings

Providing alternative models for PR goes hand-in-hand with the principle of person-centered care [13]. In the latest clinical practice guideline for PR, the recommendation is to offer either center-based PR or solutions for telerehabilitation [8]. Me&COPD includes the core components of PR—individualized exercise training, education, and behavior change strategies [13]. In addition, the results of the S-MAUQ indicated that the eHealth tool was beneficial for the participants’ health and well-being, physiotherapists’ delivery of health care to their patients, and interaction between them. Moreover, the use of cocreation with end users in the entire development process, which has been sparsely used in this field [28], further enhances the likelihood of it being a feasible eHealth tool for people with COPD. With this feasibility trial, we continue the cocreation process by letting the participants answer structured questions regarding the functionality, implications, and potential areas of improvement, which is a recommended strategy in cocreation research [31].

Previous home-based telerehabilitation programs for PR have been heterogeneous in design and content but have often included components of education and exercise [18]. However, interaction with HCPs via a web-based platform or smartphone app, as in Me&COPD, is not commonly used because communication has often been performed by videoconferencing or telephone [18]. The interactive web-based PR intervention reported by Chaplin et al [57], which was found to be a feasible alternative to conventional PR, shares some of the features of Me&COPD, that is, it was home-based and included components of individually adapted education and an exercise program with regular HCP support to progress the exercise. In PR, it is essential that the exercise program is individually prescribed and progressed [13]. In this trial, we used an exercise diary for participants to register their exercise experience and symptoms, including dyspnea and muscle fatigue ratings, after every exercise session. The purpose of the diary was to monitor exercise adherence and provide comprehensive information to the physiotherapists, enabling adjustment and progression of the participants’ exercise programs accordingly. Unfortunately, only a few sessions were registered. Participants and participating physiotherapists reported that the registration was too challenging, leading to underuse. Nevertheless, we received indications that participants exercised more frequently than the registered average of 11 (SD 10) sessions. Because of the low number of exercise registrations, the information provided to the physiotherapists to guide exercise progression was limited, and we did not find it useful to evaluate exercise intensity and progression. We believe that this was a very important result, which highlights that the design of the exercise diary was not optimal and needs further development before the eHealth tool can be implemented in clinical practice.

Me&COPD was built on the 1177 Support and Treatment platform, which is associated with advantages and disadvantages. Positive aspects are that the platform is well-known by citizens in Sweden and that several health care regions already use the platform to provide various digital health care interventions, demonstrating continuity. Furthermore, the platform is considered to be credible with high data security, which has previously been voiced to be an important criterion for an eHealth tool by prospective users [21] and by participants in this study. One of the disadvantages that was reported by participants in this study was the inconvenience of having to log in to the platform to access Me&COPD and to be able to read eventual messages from their physiotherapist. Another disadvantage was that the eHealth tool had to be adapted to the platform’s preset features and functionalities, which limited the possibility of making changes to the interface. Consequently, during its development, some functionalities, such as exercise reminders, could not be designed as intended. These concerns were validated by the participants’ feedback, which frequently highlighted difficulties in navigating the interface and locating the desired content. This aligns with previous research reporting that people with COPD prefer eHealth tools that are not overly technical and are easy to navigate [21].

The recruitment of physiotherapists and people with COPD through PHCs was challenging. We prolonged the trial, included an additional health care region, and hired a person to work specifically with the recruitment. Despite that, the intended sample size of 30 participants was not reached. Possible reasons for the low recruitment of physiotherapists might be the heavy workload in Swedish primary health care, partly related to the COVID-19 pandemic. The pandemic had a huge negative effect on the health care given to people with asthma and COPD in Sweden [58]. Therefore, people with COPD were not referred as frequently to annual follow-ups, which are important for identifying their rehabilitation needs. This was confirmed by the PHC managers and the physiotherapists during the study period, as they reported fewer referrals for PR than before the COVID-19 pandemic. Another contributing factor to the difficulty of recruiting PHCs was the demand to have the 1177 Support and Treatment platform implemented in their work organization to be included in the trial, which turned out to be a barrier in one of the health care regions. Furthermore, the views and beliefs of the HCPs can significantly influence the implementation of an innovation [20]. For example, Slevin et al [59] reported that HCPs may be unwilling to adopt eHealth solutions because there is no strong evidence base for their effectiveness. Due to this, they may not recommend such treatment options to their patients, which could have affected the recruitment in our study as well. Other known barriers from the literature regarding the implementation of eHealth tools are a lack of organizational support to change the work procedures and insufficient digital literacy among the HCPs [59,60]. A possible barrier to the recruitment of people with COPD might have been that some were skeptical to replace a face-to-face contact with an HCP with an eHealth solution, as previously described in the literature [21,61] and by physiotherapists within this trial. By contrast, only 2 (5%) of the 39 eligible participants for this trial indicated that their main reason for declining participation was a preference for face-to-face treatment. Nevertheless, although our sample size fairly aligned with the recommendations for pilot trials [56], we have realized that the procedure of recruitment needs some consideration to be a feasible alternative in a larger trial.

Clearly, eHealth solutions are not universally suitable, despite the high level of internet access and digital engagement among people with COPD in Sweden [62]. Participants in this trial expressed that some degree of computer skills was required to manage the eHealth tool. To provide additional support and the possibility of repeated information, a written user manual, similar to the one that the physiotherapists received, could be considered, along with incorporating a film within the eHealth tool describing the features of the intervention. Consequently, digital literacy should be considered in clinical practice when choosing the best treatment for the patient [57]. Applying a patient-centered approach where aspects such as perceived usefulness, digital literacy, self-efficacy, and social context are considered has been suggested to be important when it comes to the individual’s readiness to adopt a digital health intervention [61]. However, in the end, the individual’s treatment preference may be the most important for the completion of an intervention.

Strengths of this trial include its design as a multicenter randomized controlled trial and the use of cocreation in both the development process and throughout the study. The eHealth tool seemed safe to use, and the exercises included in the program were both feasible and suitable for a home environment, requiring no equipment besides resistance bands. Furthermore, the pragmatic design, that is, the trial being conducted as part of ordinary clinical practice, increases the chances of a successful future implementation.

### Limitations

To evaluate usability, we used a Swedish version of the MAUQ questionnaire. The original questionnaire was developed for evaluating mobile health (mHealth) apps [42]. However, it is important to note that our eHealth tool is not solely an mHealth app, as the 1177 Support and Treatment platform, through which Me&COPD is accessed, is also available via a website. The original questionnaire has been found to have good validity and reliability, although the HCP versions need further evaluation [42]. We used a structured process when translating the questionnaire into Swedish, but no psychometric analysis of the Swedish version has been performed, which may be acknowledged as a limitation. The MAUQ has no established cutoff or reference values for what is considered as good usability. A possible reason for this may be that the questionnaire has not often been used in mHealth studies aiming to evaluate usability, despite that it was specifically designed to evaluate that outcome [63]. Nevertheless, this makes it a bit difficult to interpret the results. A previous study using the stand-alone version of MAUQ for patients suggested that an average score <4 would indicate that the usability is not good [64]. Comparable to the approach in this study, previous studies using the interactive versions of the questionnaire have chosen to report the average overall score and the scores for respective subscales with reference to the descriptions of each number on the 7-point Likert scale [65,66]. A couple of the statements in the health care version received a low response rate because the physiotherapists had chosen the “not applicable” option. The first statement concerned the use of the eHealth tool in social settings, which was not relevant for them, and the other question was concerned with whether it had been easy to recover from making a mistake in the app. Choosing the “not applicable” option in this case could be considered positive if it meant that they had not made any mistakes when using the eHealth tool.

One limitation was that we did not measure physiotherapist adherence, which means that we do not know if they complied with the intervention’s recommended work procedures. Furthermore, due to the nature of this feasibility trial, no statistical between-group comparisons were performed [35], and no clear trends in the clinical outcomes were seen. The intervention was delivered as a complement to the health care provided for people with COPD, primarily within primary care. The participants in both groups could take part in other interventions during the trial, which could hypothetically make it harder to evaluate the effect of a specific intervention. However, only 21% (4/19) of the participants, 23% (3/13) in the intervention group and 17% (1/6) in the control group, declared that they had received additional COPD-related health care contacts, of which most of them were single follow-ups. Only 17% (1/6) of the participants in the control group participated in a PR program at an outpatient health care facility, which supports the notion that access to PR is limited in people with COPD.

### Conclusions

The novel eHealth tool, Me&COPD, seems to be a safe and feasible alternative strategy for PR because the tool was found to have acceptable usability by people with COPD as well as participating physiotherapists. Usability may be improved by better organization of the information and simplification of the exercise diary to enable collection of data on exercise adherence, intensity, and progression via the eHealth tool. The test procedures and outcome measures used in the trial worked satisfactorily, but the recruitment process needs further consideration. The results from this trial can be used to inform the design of future research studies with similar interventions, although the effectiveness of the intervention remains to be evaluated in a future larger trial. When the tool has been revised and is ready to be implemented in clinical practice, the intervention can be offered to people with COPD across the country, thanks to the established nationwide digital health care platform.

## Appendix

Multimedia Appendix 1 CONSORT-EHEALTH (Consolidated Standards of Reporting Trials of Electronic and Mobile Health Applications and Online Telehealth) checklist (V 1.6).

## Abbreviations

CONSORT: Consolidated Standards of Reporting Trials
COPD: chronic obstructive pulmonary disease
HCP: health care provider
MAUQ: Mobile Health App Usability Questionnaire
mHealth: mobile health
PHC: primary health care center
PR: pulmonary rehabilitation
S-MAUQ: Swedish version of the Mobile Health App Usability Questionnaire
TIDieR: Template for Intervention Description and Replication

## Data Availability

The datasets generated or analyzed during this study are available from the corresponding author on reasonable request.
